# Isoniazid Causing Drug-Induced Thrombocytopenia

**Published:** 2011-06-08

**Authors:** Donald R Laub

**Affiliations:** University of Vermont College of Medicine, Burlington, VT

## DESCRIPTION

DW was a born in China with complete unilateral cleft lip and palate. She was placed in an orphanage shortly after birth. At 19 months of age, a family in the United States adopted her, and shortly thereafter, she underwent uneventful cleft lip repair. At 20 months of age she had a positive Mantoux Purified Protein Derivative (PPD) test and was begun on Isoniazid monotherapy for possible latent tuberculosis. She was brought to the operating room a second time at 22 months of age for a palatoplasty. She was noted to have abnormal bleeding intraoperatively. Her platelet count was found to be 43,000 per microliter; she was diagnosed as having drug-induced thrombocytopenia (DITP). Her palatoplasty was completed, albeit with less extensive dissection that might otherwise have been performed. She was admitted to the Pediatric Intensive Care Unit, intubated and had transfusion with platelets and packed red blood cells. She did well with clinical resolution of hemorrhage and was extubated. She postoperatively developed a palatal fistula. In the perioperative period her Isoniazid therapy was held, and her platelet count recovered to 203,000 per microliter. She subsequently had a positive antigen test for mycobacteria, confirming a latent tuberculosis infection. She was begun on Isoniazid once again. She completed 9-month course of Isoniazid, during which time her platelet count reached a second nadir of 107,000 per microliter; she had no further episodes of abnormal bleeding. At 28 months of age, after completion of her antitubercular therapy, her platelet count recovered to 363,000 per microliter. She underwent revision palatoplasty, uneventfully, which was successful in closing her palatal fistula.

## QUESTIONS

1. **How should a surgeon evaluate a possible case of DITP?**
2. **Where can a surgeon find a database of DITP reports?**
3. **What is the process for reporting a case of DITP?**

## DISCUSSION

The most widely accepted model for DITP postulates preexisting antibodies with low affinity to platelet epitopes; in the presence of an inducing drug, these are converted to high affinity to this epitope.^1^^-^6

 The University of Oklahoma Health Sciences Center maintains a Web-based database of case reports of DITP; this database lists 1299 single patient reports, and 143 group series reports collected up to October 20, 2010.^7^ There is a single case report by Hansen in 1961 of Isoniazid causing DITP.^8^

 George et al^9^ published a systemic review of published case reports of DITP. In this review, they set forth criteria for evaluation of published patient data:

1. Drug administration preceded thrombocytopenia; recovery from thrombocytopenia completed and sustained after drug discontinued.
2. Other drugs administered prior to thrombocytopenia were continued or reintroduced after discontinuation of the suspected drug.
3. Other etiologies of thrombocytopenia excluded.
4. Reexposure to the drug resulted in recurrent thrombocytopenia.

On the basis of these criteria, they stratified studies into the following levels of evidence:

1. Definite: all 4 criteria met.
2. Probable: criteria 1-3 met.
3. Possible: criterion 1 met.

George et al^9^ then tallied the case reports and found 48 drugs with level 1, or definite evidence, and 15 drugs with only level 2 or probable evidence of causing thrombocytopenia. Hansen's 1961 report of DITP from Isoniazid met the criteria for level 1 or definite evidence for DITP.^8^ The 21 drugs with 5 or more reports that meet either definite or probable evidence are presented in tabular form (Table 1).

 The patient presented in this case report shows level 1 evidence of DITP based on the criteria described by George et al:

1. Administration of Isoniazid preceded the episode of thrombocytopenia, and recovery was sustained after withdrawal of the drug, both initially, and after reintroduction.
2. There were no other drugs, other than the anesthetic agents and prophylactic antibiotics administered during the surgical procedure. The time course of the bleeding episode makes these very unlikely as causative agents for DITP.
3. The child did not show hemolytic anemia, nor progress to uremia or neurologic sequella, does not meet the diagnostic criteria of thrombotic thrombocytopenic purpura-hemolytic uremic syndrome.^10^ Likewise, she showed resolution of her thrombocytopenia, so diagnosis of idiopathic thrombocytopenia purpura would not be accurate.^11^
4. Her Platelet count dropped again after resumption of Isoniazid, albeit without clinical consequences.

George et al offers the following a protocol for evaluation of patients with suspected DITP:

1. Determine the probability for a causal relation of the suspected drug to the occurrence of thrombocytopenia using the clinical criteria presented in Table 1.
2. Determine previously published experience.
3. Check the DITP database (www.ouhsc.edu/platelets) for previously published reports describing the suspected drug. Are there previous reports with definite or probable evidence for a causal relation of the suspected drug and thrombocytopenia?
4. Determine the presence of drug-dependent antiplatelet antibodies to confirm the etiologic role of the drug that is suspected as the cause of thrombocytopenia.
5. If the drug is confirmed as the cause of thrombocytopenia by clinical criteria and/or by demonstration of drug-dependent antiplatelet antibodies, report the patient to the Food and Drug Administration Adverse Event Reporting System by using www.fda.gov/medwatch/.
6. If there are no or few published case reports with definite or probable clinical evidence for a causal role of the drug with thrombocytopenia, publish your experience.

 This is the second case report of Isoniazid-induced thrombocytopenia in 50 years; it seems unlikely that another case will be seen by a practicing plastic surgeon with any frequency. It is instructive, however, to review the process for evaluation of possible cases of DITP, as well as the protocol for reporting index cases.

## Figures and Tables

**Figure 1:**
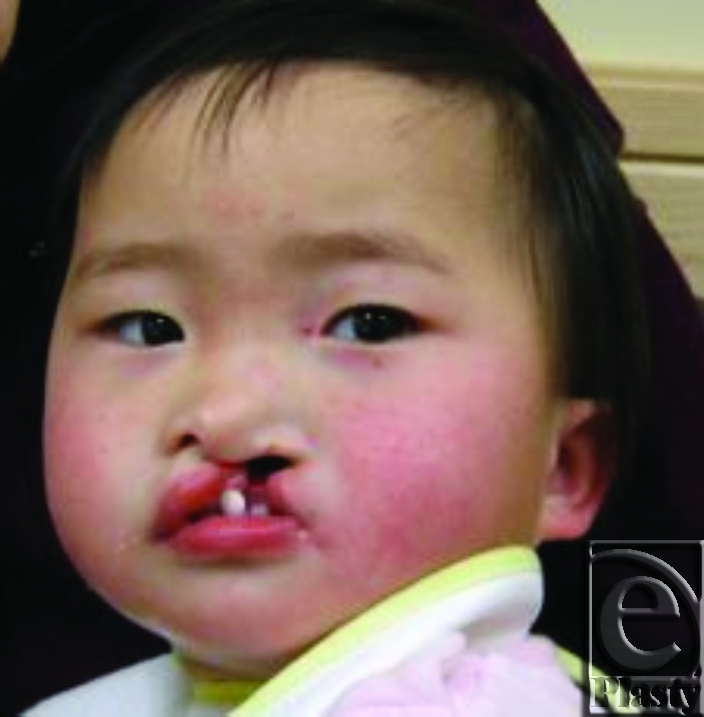


**Table 1:** Drugs likely to cause thrombocytopenia (definite or probable evidence), after George et al^9^

	Number of Reports	
Drug (Brand Name)	Definite Evidence	Probable Evidence
Abciximab (ReoPro)	6	7
Acetaminophen (Tylenol, Panadol)	3	4
Carbamezapine (Tegretol)	0	10
Chlorpropamide (Diabinese)	0	5
Cimetidine (Tagamet)	1	5
Danazol (Danocrine)	3	4
Diclofenac (Cataflam and Voltaren)	2	3
Efalizumab (Raptiva)	0	6
Eptifibatide (Integrilin)	2	7
Gold (Ridaura, Solganal)	0	11
Hydrochlorothiazide (Diuril)	0	5
Interferon-α (Roferon-A)	1	6
Methyldopa (Aldomet)	3	3
Nalidixic Acid (NegGram)	1	5
Quinidine (Quinaglute, Cardioquin)	26	32
Quinine (Quinamm, Quindan)	14	10
Ranitidine (Zantac)	0	5
Rifampin (Rifadin, Rimactane)	5	5
Tirofiban (Aggrestat)	2	6
Trimethoprim/sulfamethoxazole (Bactrim, Septra)	3	12
Vancomycin (Vancoled)	3	4
